# Evaluating medication-related quality of care in residential aged care: a systematic review

**DOI:** 10.1186/s40064-015-0984-9

**Published:** 2015-05-14

**Authors:** Jodie B Hillen, Agnes Vitry, Gillian E Caughey

**Affiliations:** University of South Australia, Quality use of medicines and pharmacy research centre (QUMPRC), School of Pharmacy and Medical Sciences, University of South Australia, Adelaide, Australia

**Keywords:** Aged or aged, Over 80, Residential aged care or nursing home, Quality improvement, Quality indicators, Medication safety and Systematic review

## Abstract

Given the growing aged care population, the complexity of their medication-related needs and increased risk of adverse drug events, there is a necessity to systematically monitor and manage medication-related quality of care. The aim of this systematic review was to identify and synthesise medication-related quality of care indicators with respect to application to residential aged care. MEDLINE (Ovid), Psychinfo, CINAHL, Embase and Google® were searched from 2001 to 2013 for studies that were in English, focused on older people aged 65+ years and discussed the development, application or validation of original medication-related quality of care indicators. The quality of selected articles was appraised using the Critical Appraisal Skills Program and psychometric qualities extracted and synthesised using content analysis. Indicators were mapped to six medication-related quality of care attributes and a minimum indicator set derived. Thirty three articles describing 25 indicator sets met the inclusion criteria. Thirteen (52%) contained prescribing quality indicators only. Eight (32%) were developed specifically for aged care. Twenty three (92%) were validated and seven (28%) assessed for reliability. The most common attribute addressed was medication appropriateness (n = 24). There were no indicators for evaluating medication use in those with limited life expectancy, which resulted in only five of the six attributes being addressed. The developed minimum indicator set contains 28 indicators representing 22 of 25 identified indicator sets. Whilst a wide variety of validated indicator sets exist, none addressed all aspects of medication-related quality of care pertinent to residential aged care. The minimum indicator set is intended as a foundation for comprehensively evaluating medication-related quality of care in this setting. Future work should focus on bridging identified gaps.

## Key points

- **Twenty eight previously validated medication-related quality of care indicators (minimum indicator set) were identified as relevant to both the aged care setting and clinical needs of residents.**
- **Validated indicators which address appropriate medication-related care in those with limited life expectancy are lacking and need to be explored further.**
- **The minimum indicator set developed in this study is intended as a foundation for increasing the utilisation of medication-related quality of care indicators in the aged care sector.**

## Introduction

In Australia and other developed countries the population is ageing rapidly. Since 1970, the Australian population has aged significantly with a six-fold increase in the proportion of adults aged 85 years and older (Australian Institute of Health and Welfare 2010) with similar statistics reported in the UK, Europe and the USA (Office for National Statistics. Statistical Bulletin: Older People’s Day 2011; Howden & Meyer 2011). One consequence of an ageing population is increased demand on health care and social support systems. In 2011, 165,000 Australians lived in permanent aged care (Australian Institute of Health and Welfare 2012) and it is projected that by 2050 aged care services will be provided to around 3.5 million Australians (1 million in permanent residential care) at a financial cost of $1.8% of the GDP (Commonwealth of Australia 2010).

The prevalence of multiple chronic conditions (multimorbidity) in the older population is reported to be between 65 to 80% (Britt et al. 2008). Multimorbidity is associated with use of multiple medicines (polypharmacy) which in turn is linked to medication-related adverse outcomes including falls, death and hospitalisation (Wilson et al. 2010; Milton et al. 2008). This is compounded by the physiologic effects of ageing, such as altered clearance of medicines (Milton et al. 2008). In the United States medication-related adverse events in residential aged care have been estimated to be between 1.19 to 7.26 incidents per 100 resident-months and for every $1 (US) spent on medicines in aged care it is estimated $1.33 is spent on treating adverse events (Bootman et al. 1997).

Given the growing aged care population, the complexity of their medication-related needs and increased risk of adverse drug events, there is a necessity to systematically monitor and manage medication-related quality of care. Quality of care is traditionally evaluated using indicators which target the most relevant aspects of healthcare (Arah et al. 2006; Mainz 2003). Whilst there is ongoing constructive debate regarding the relationship between quality of care indicators and patient outcomes, they remain the gold standard for evaluating the quality of many aspects of health care. For example, the National Committee for Quality Assurance in the USA and the National Institute for Health and Care Excellence in the United Kingdom both advocate the use indicators for evaluating quality of care (National Committee for Quality Assurance & USA: www.ncqa.org Accessed [September 2013; National Institute for Health and Care Excellence 2014a). In the Australian setting, the 2011 Productivity Commission Inquiry Report, *Caring for Older Australians*, identified the need for validated indicators that address the quality of care in the aged care population, including medication-related quality of care (Productivity Commission 2011).

Whilst a variety of indicators exist to evaluate the medication-related quality of care in the ambulatory elderly, few specifically address the needs of the residential aged care population. The aged care population is commonly frailer and more dependent than the ambulatory older population and as a consequence has different medication-related needs. Expectations of health outcomes may also differ between the two populations due to differences in quality of life values and life expectancy. Additionally, many medication-related quality of care sets are overburdened with dozens of indicators and minimal direction for prioritising the evaluation process, minimizing their utilisation in the clinical setting.

The aims of this systematic review were to identify, describe and consolidate the most appropriate and feasibly actionable medication-related quality of care indicators for use in residential aged care and more specifically for Australian residential aged care.

## Methods

### Data sources

MEDLINE (Ovid), PsycINFO, Embase and CINAHL databases were searched between January 2001 to December 2013. The following search terms (Medical Subject Headings and keywords) were used for all four databases: Quality Assurance, Health Care/ or *“Quality of Health Care”/ or “quality of health care*”.mp. AND*Quality Indicators, Health Care/ or *“Outcome and Process Assessment (Health Care)”/ or clinical indicator*.mp. OR prescribing indicator*.mp. OR *Polypharmacy/ or polypharmacy*.mp. OR inappropriate prescribing*.mp. OR “Quality use of medicine*”.mp. The search was limited to articles published in English and focusing on older people aged 65 years and older. Reference lists of eligible papers were searched to further identify suitable publications. If an article identified via reference check list was not published within the pre-defined time frame it was included if the indicator(s) was currently used in public reporting or research. ‘Quality in Health care’ was used as the search term for Google® with the first 100 hits screened to maximize relevance to the search criteria. Authors’ knowledge of government and organisation websites with a focus on delivery and/or evaluation of health care were also searched for relevant articles (Websites searched include AIHW (Australian Institute of Health and Welfare) (www.aihw.gov.au), Institute for Healthcare Improvement (www.ihi.org), NPS (National Prescribing Service) (www.nps.org.au), European Directorate for the Quality Use of Medicines & Healthcare (www.edqm.eu), European Society for Quality in Health care (www.esqh.net), CMS (Centers for Medicare and Medicaid Services) (www.cms.gov), Health Indicators Warehouse (www.healthindicators.gov), NICE (National Institute for Health and Clinical Excellence) (www.nice.org.uk), RAND Corporation (www.rand.org), Cochrane Collaboration (www.cochrane.org) and Emerging Researchers in Ageing (www.era.edu.au).

The search strategy was developed in consultation with a librarian specialising in health databases. Methods for identifying and selecting the articles were predetermined in a protocol developed collaboratively by all three authors. The reporting of this systematic review conforms to the PRISMA checklist endorsed by the Cochrane Collaboration (Moher et al. 2009).

### Study selection

Original articles describing the development of an indicator or set of indicators to assess the quality of care in the elderly (>65 years) were included if they addressed at least one aspect of medication use. Articles were excluded if they: (1) were undertaken in populations with specific care needs mostly unrelated to the older population such as paediatrics, oncology, obstetrics and HIV patients (2) focused on assessing the quality of care in settings unrelated to aged care such as emergency care and surgery or (3) discussed the application of, or adaptation of an existing indicator(s). For example, articles which discussed the adaption of Beers Criteria to a country’s pharmacopeia were excluded as this was considered an adaption of an existing indicator set. Research involving the amalgamation of several indicator sets was also excluded. Articles relating to transitional care were excluded as the focus of this research is not the process of moving into or out of aged care.

For the purposes of this research a medication-related quality of care indicator was defined as a ***‘measure to be used as a guide to monitor, evaluate and improve the direct and indirect aspects of medication use affecting quality of care and patient outcomes.’*** Direct aspects of medication use include prescribing, administration of medications and clinical services related to medication use. Indirect aspects of medication use include organisational factors and health outcomes.

### Reviewers

One author (JH) conducted the initial database search, the first three sifts of articles, the Google® search and reference list check. Relevant articles were assessed for quality using the Critical Appraisal Skills Program (CASP) criteria by one author (JH) (Critical Appraisal Skills & United Kingdom: www.casp-uk.net Accessed [September 2011). All three reviewers (JH, AV and GC) met to discuss the appropriateness of inclusion of each article with respect to the inclusion criteria and the quality of the article. Disagreements were resolved by discussion. Articles were excluded if they did not meet all CASP criteria. CASP is recommended by the Cochrane Collaboration as a simple and effective tool to analyse the quality of qualitative research (Hannes 2011).

### Data extraction and synthesis

Data extraction and synthesis was undertaken in several stages. Firstly, descriptive qualities were extracted from each indicator set with an emphasis on medication use, country of origin, target population and operational status by one author (JH). Secondly, content analysis was used to synthesise the psychometric properties of the indicator sets as it is a systematic, effective and transparent method for categorising data and identifying commonalities between qualitative studies (Dixon-Woods et al. 2005). Whilst there is no standardised way of categorising quality of care indicators, several key psychometric qualities were identified from the literature (National Committee for Quality Assurance & USA: www.ncqa.org Accessed [September 2013; Clark & Bierman 2009; Australian Institute of Health and Welfare 2009). Consumer involvement was added due to its significance to Australian medicines policy (Department of Health and Ageing 1999). The final content analysis template was approved by all authors and systematically applied using a standardised data collection form by one author (JH). All authors (JH, AV and GC) met and reviewed the descriptive qualities and content analysis results for each article. Disagreements were resolved by discussion.

Thirdly, a subsequent search to establish basic operational status and further validation studies of the included indicator sets was performed by one author (JH) in February 2014 using Google® and Google scholar® with the indicator name.

### Definitions of key psychometric properties

**The aspect of health care they evaluate:** indicators can be described using a three part inter related hierarchical structure (Donabedian 1966). Structural indicators evaluate the characteristics of the organisation providing the care, process indicators evaluate the care that is provided and outcome indicators evaluate the results of the care provided.

**Type of indicator:** explicit indicators are criteria-based whereas implicit indicators rely more on individual clinical judgment (Donabedian 1988; Lund et al. 2011; Chang & Chan 2010; Spinewine et al. 2007).

**Scientific merit of the indicators**: indicators should measure what they intend to measure (content validity), be meaningful and relevant to the key audience (face validity) and identify the same effect when measured at different times or by different people (reliability) (Martirosyan et al. 2010). Other properties that are useful to demonstrate are concurrent validity (comparison of indicator to a gold standard) and predictive validity (the ability of the indicator to predict a health outcome) (Martirosyan et al. 2010).

**Real life application:** both external validity (the ability of the indicator to be applied as effectively to another health care setting or in another country) and feasibility (the degree to which the required data can be collected and reported in a timely manner) describe the practicality of the indicator and can often determine if the indicator is reported (National Committee for Quality Assurance & USA: www.ncqa.org Accessed [September 2013; Burchett et al. 2011).

**Consumer involvement:** consumers are central to Australia’s National Medicines Policy (Department of Health and Ageing 1999) and should contribute to the discussion on prioritizing measures to evaluate aspects of health care rather than relying solely on expert opinion.

Once identified and described each indicator set was mapped to six of the most relevant medication-related quality of care attributes for Australian residential aged care. These attributes were derived by all authors (JH, AV and GC) from sentinel papers on quality of care for the medically complex elderly, known epidemiology of chronic disease and medication use in Australian residential aged care and Australian medicines policy. The initial mapping was undertaken by one author (JH) and results were reviewed by two authors (AV and GC). These attributes do not address every medication-related quality of care issue for aged care residents. They are intended to assist with targeting the most significant issues according to the available information at the time of this study. They are intended to focus evaluation of medication-related QOC on the greatest burden of disease, greatest medication-related risk factors and relevant quality of care activities in the aged care population. This parsimonious approach to selecting indicators is concordant with the conceptual framework for developing and implementing health care indicators as it improves the feasibility of implementation (Donabedian 1980).

### Medication-related attributes relevant to Australian residential aged care

**General medication appropriateness:** Appropriateness of medication is a central tenant to many quality of care models including the Australian quality use of medicines strategic plan (Australian Institute of Health and Welfare 2009; Department of Health and Ageing 1999; Donabedian 1980). Medication appropriateness is defined as the use of a medication only when necessary, with consideration to an individual’s clinical indications, co-existing conditions and the risk-benefit profile using evidenced-based criteria.

There is a growing awareness of the complexity between managing patient’s multiple chronic diseases using evidence based guidelines for each condition and maximising the benefit to the individual (Tinetti et al. 2004; Tinetti et al. 2012; Gilbert et al. 2011). This is particularly relevant to the challenges of assessing quality of care in the frail and medically complex older population, who are commonly excluded from clinical trials from which evidence based guidelines are derived. (Holmes et al. 2013; Marengoni 2013). Attention will be directed to identifying indicators which attempt to address this gap.

**Medication appropriateness for the most prevalent chronic diseases:** The most prevalent diseases in the Australian aged care population are dementia, cardiovascular disease (CVD) and musculoskeletal conditions (Australian Institute of Health and Welfare 2012).

**Medication appropriateness those with limited life expectancy:** The majority of Australian aged care residents die within three years of admission to residential care (Australian Institute of Health and Welfare 2012). Medication should be reconciled with respect to time to benefit (Holmes et al. 2013).

**Detection and monitoring for adverse drug events:** Chart review studies undertaken in Australian residential aged care reveal that the majority of residents are exposed to over five medicines per day many of which are associated with adverse outcomes in the older population (Somers et al. 2010; Wilson et al. 2011). In 2008, a population based study found that residents were taking on average nine or more medicines with 50% exposed to psychotropic medication which are known to be related to adverse events such as falls (Roughead et al. 2008). Attention will be directed to indicators which monitor and detect for adverse drug events.

**Access to medication-related services:** Australian aged care residents are entitled to several medication-related services, such as medication reviews, which may influence the quality of medication use (Gilbert et al. 2002). These services are an opportunity to modify prescribing according to the resident’s current clinical condition as well as identifying adverse drug reactions.

**Medication-related quality of care policies:** Both in Australia and overseas, it is recommended that residential aged care facilities regularly review medication management policies and procedures as part of their quality improvement cycle (Department of Health 2012; National Institute for Health and Care Excellence 2014b).

### Developing the minimum indicator set

The results from mapping were used to guide the development of a minimum indicator set for evaluating medication-related quality of care in Australian residential aged care. The objectives were to identify and synthesise the most appropriate indicators with respect to the core needs of this population (six key medication-related attributes) which can be feasibly implemented within the Australian aged care setting to facilitate the assessment of quality of care.

Individual indicators were included in the minimum indicator set if they addressed at least one of the six core medication-related attributes and were present in three or more of the identified indicator sets. This represented the most parsimonious balance of addressing the majority of attributes without overburdening the evaluation process. Indicators included in the minimum indicator set also needed to be feasibly collected and reported at a population level. The minimum indicator set was initially developed by one author (JH) and reviewed by two authors (AV and GC) and disagreements resolved by discussion.

## Results

**Figure 1:**
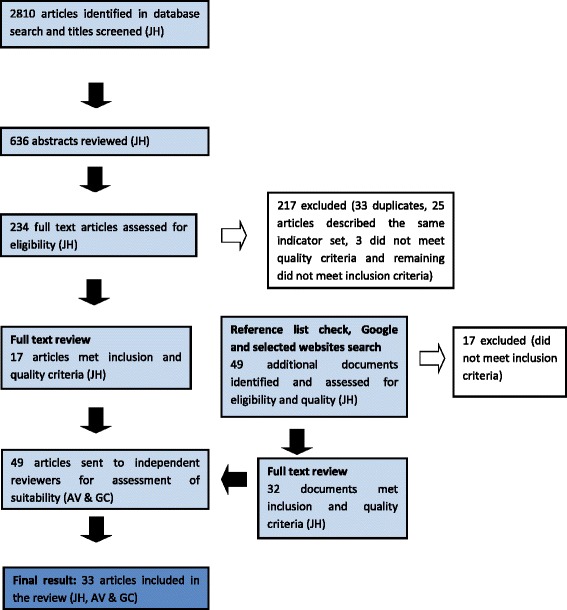
Flowchart of the study selection process.

A total of 33 articles discussing 25 independent indicator sets were included in the systematic review (see Figure 1 for study selection flowchart). For some indicator sets more than one article was necessary to describe their development. Table 1 describes the included indicator sets grouped according to their emphasis on medication use and described by country of origin, target population and current operational status.

**Table 1:** **Characteristics of identified indicator sets (Country of origin)**

**Name of indicator/indicator set (n = 25)**	**Target population**	**Brief description**	**Operational status**
**Quality of care indicator sets with at least one medication-related quality of care indicator (n = 8)**			
Resident-centred quality indicators in residential aged care or *The Campbell Report* (Campbell Research and Consulting (CR&C) (Campbell Research and Consulting (CR&C) 2006))	Aged care	24 indicators. ‘Prevalence of medication use’ is the only medication-related indicator.	Not piloted and not operational (National Aged Care (National Aged Care Alliance 2014))
(Australia)			
Public Sector Residential Aged Care Quality of Care Performance Indicators (Nay et al. 2004)	Aged care	6 indicators. Polypharmacy (9 or more medications) is the only medication-related indicator.	The set has been introduced across the whole of public service residential aged care in Victoria. No external reporting (National Aged Care (National Aged Care Alliance 2014)).
(Australia)			
ResCareQA (Residential Care Quality Assessment formally the Clinical Care Indicators Tool or CCI) (Courtney et al. 2007; Courtney et al. 2011)	Aged care	23 indicators of which two are medication related. Polypharmacy (9 or more medications) and medication review.	Piloted (National Aged Care (National Aged Care Alliance 2014)) and assessed for content validity (Courtney et al. 2011).
(Australia)			
National indicators of safety and quality in health care (Australian Institute of Health and Welfare 2009)	All health care sectors	55 indicators of which 5 relate to aged care and 1 to medication (annual medication review). 22 indicators with potential to evaluate medication-related quality of care in aged care such as pharmacological management of hypertension.	Not operational. Information relating to some of the indicators is available in various government reports.
(Australia)			
Minimum Data Set (version 3.0) Nursing Home Quality Measures.(Centres for Medicare and Medicaid Services & USA: www.cms.gov Accessed [February 2014; Zimmerman et al. 1995; Hawes et al. 1997)	Aged care	18 measures covering several aspects of aged care (derived from Resident Assessment Instrument-MDS developed in 1995).Medication-related indicators include vaccination rates and use of antipsychotic medications.	Mandated quarterly reporting of indicators on Centres for Medicare and Medicaid website. Updated regularly.
(USA)			
Assessing Care of Vulnerable Elders (version 3) or ACOVE–3 (Wegner et al. 2007)	Aged care and ambulatory elderly	392 indicators covering 26 conditions. Medication use addressed by 98 indicators including mediation specific indicators, medication review rates, continuity of care and medication list reconciliation.	Extensively reported in the scientific literature and used as a model for adaptations in other countries. Studies and reports available online.
(USA)			
Healthcare effectiveness data and information set or HEDIS (National Committee for Quality Assurance & USA: www.ncqa.org Accessed [September 2013)	All health care sectors	83 indicators of which approximately one third are medication-related indicators including condition specific (e.g. treatment of COPD, asthma and diabetes) and general indicators (e.g. medication review, post-discharge medication reconciliation and medications to avoid in the elderly (Beer’s criteria)).	Approximately 90% of US health plans report this data voluntarily. Selected reports available online. Updated annually.
(USA)			
Quality and Outcomes Framework or QOF (Primary Care & Social Care Information Centre 2013)	General practice	Over 140 indicators. Clinical indicators cover 22 clinical areas and contain many medication related indicators (e.g. appropriate treatment of hypertension and medication review).	Voluntary annual reporting by General Practice with pay-for-performance incentives. Results available online. Updated annually.
(UK)			
**Indicator sets with a primary focus on medication-related quality of care (n = 4)**			
Guiding principles for medication management in residential aged care facilities (Australia) (Department of Health 2012)	Aged care	17 guiding principles covering governance, prescribing, medication administration, medication storage and evaluating practice.	Released 2012. Not operational.
Indicators for Quality Use of Medicines in Australian Hospitals (NSW Therapeutic Assessment Group 2007) (Australia)	Hospital	30 indicators covering prescribing, medication monitoring and medicine education.	Not operational.
Indicators for Quality Prescribing in Australian General Practice (National Prescribing Service 2006) (Australia)	General practice	21 indicators covering prescribing, monitoring, education and review of medications.	Not operational.
Preventable Drug Related Morbidity (*PDRM)* (Mackinnon & Hepler 2002; Robertson & MacKinnon 2002; Morris et al. 2002) (Canada and USA)	Geriatric	52 indicators identifying health care utilisation due to inappropriate or failure to use medications. Each indicator explicitly states a pattern of care and the resulting outcome.	Reported in scientific literature.
**Indicator sets addressing prescribing (PQIs) (n = 13)**			
Australian Prescribing Indicators Tool (Basger et al. 2008) (Australia)	General Practice	48 prescribing indicators for patients 65 years or older. Focus is on drug-drug and drug-disease interactions.	Reported in scientific literature.
Drug Burden Index or DBI (Hilmer et al. 2007)	General Practice	A patented formula for calculating the total sedative and anticholinergic load in an individual.	Reported in scientific literature.
(USA)			
The PRISCUS List (Holt et al. 2010)	Geriatric Prescribing	83 potentially inappropriate medications in the elderly (> = 65 years) with recommendations and alternatives.	Reported in scientific literature.
(Germany)			
Inappropriate Prescribing in the Elderly Tool or IPET (McLeod et al. 1997; Naugler et al. 2000)	Hospital	Originally the McLeod criteria, adapted in 2000 to the IPET which has 14 inappropriate prescribing indicators.	Reported in scientific literature.
(Canada)			
Beer’s criteria (The American Geriatrics Society 2012)	Aged care and ambulatory care	Original set of indicators from 1991, last updated 2012. 53 recommendations for medications to be avoided in the elderly (> = 65) or avoided in elderly with certain conditions/medications.	Reported extensively in the scientific literature and used in the HEDIS dataset (Marcum & Hanlon 2012).
(USA)			
Medication Appropriateness Index or MAI (Hanlon et al. 1992)	General Practice	Classifies appropriateness of each medication against ten criteria.	Reported in scientific literature.
(USA)			
NORGEP criteria for assessing inappropriate prescriptions to elderly patients (Rognstad et al. 2009)	General Practice	36 criteria assessing use of particular medications and drug combinations in the 70+ population.	Reported in scientific literature.
(Norway)			
Criteria for drug selection in frail elderly patients (Huisman-Baron et al. 2011)	Frail elderly	23 criteria to assess individual drug classes in the frail elderly.	Reported in scientific literature.
(Netherlands)			
The Screening tool of Older Person’s Prescriptions (STOPP) and Screening Tool to Alert to Right Treatment (START) Criteria	Hospital and general practice	22 START (address under prescribing) and 65 STOPP (address inappropriate prescribing) criteria.	Extensively reported in the literature and currently used in an international database trial (The SENATOR Project & Europe: www.senator-project.eu Accessed [February 2014).
(O'Mahony et al. 2010; Barry et al. 2007; Gallagher & O'Mahony 2008)			
(Ireland)			
Criteria for high-risk medication use (Winit-Watjana et al. 2008)	Elderly	77 indicators to assess prescribing quality in the elderly.	Not operational
(Thailand)			
Potentially inappropriate medications in elderly: a French consensus panel (Laroche et al. 2007)	> = 75 years	36 indicators covering medications to avoid and medications to avoid in certain conditions in the elderly.	Reported in scientific literature
(France)			
Potentially inappropriate prescriptions for older patients in long-term care or PIP (Rancourt et al. 2004)	Aged care	111 prescribing indicators covering inappropriate medication, duration, dosage and medication combinations.	Reported in scientific literature
(Canada)			
CRIteria to assess appropriate Medication use among Elderly complex patients (CRIME) (Onder et al. 2013)	Clinically complex elderly	19 recommendations addressing treating older complex patients with at least one of the following chronic disease: diabetes, hypertension, congestive heart failure, atrial fibrillation and coronary artery disease.	Currently undergoing validation for clinical outcomes.
(Italy)			

Eight of the identified indicator sets are general quality of care indicator sets which contain at least one medication-related quality of care indicator (National Committee for Quality Assurance & USA: www.ncqa.org Accessed [September 2013; Australian Institute of Health and Welfare 2009; Campbell Research and Consulting (CR&C) 2006; Nay et al. 2004; Courtney et al. 2007; Centres for Medicare and Medicaid Services & USA: www.cms.gov Accessed [February 2014; Wegner et al. 2007; Primary Care & Social Care Information Centre 2013). Four indicator sets focus on medication-related quality of care and evaluate a range of activities related to medication use (Department of Health 2012; NSW Therapeutic Assessment Group 2007; National Prescribing Service 2006; Mackinnon & Hepler 2002). Thirteen sets address prescribing and these are commonly referred to in the literature as prescribing quality indicators or PQIs (Basger et al. 2008; Hilmer et al. 2007; Holt et al. 2010; McLeod et al. 1997; The American Geriatrics Society 2012; Hanlon et al. 1992; Rognstad et al. 2009; Huisman-Baron et al. 2011; O'Mahony et al. 2010; Winit-Watjana et al. 2008; Laroche et al. 2007; Rancourt et al. 2004; Onder et al. 2013). The majority of indicator sets originated from Australia (n = 8) (Australian Institute of Health and Welfare 2009; Department of Health 2012; Campbell Research and Consulting (CR&C) 2006; Nay et al. 2004; Courtney et al. 2007; NSW Therapeutic Assessment Group 2007; National Prescribing Service 2006; Basger et al. 2008) and the USA (n = 6) (National Committee for Quality Assurance & USA: www.ncqa.org Accessed [September 2013; Centres for Medicare and Medicaid Services & USA: www.cms.gov Accessed [February 2014; Wegner et al. 2007; Hilmer et al. 2007; The American Geriatrics Society 2012; Hanlon et al. 1992).

Eight of the indicator sets were developed specifically for aged care. Two of these eight addressed several aspects of medication-related quality of care (e.g. prescribing, medication review, consumer counseling, medication reconciliation) (Department of Health 2012; Wegner et al. 2007). Three included one or more of the following medication-related quality of care indicators: polypharmacy, prevalence of medication use and medication review (Campbell Research and Consulting (CR&C) (Campbell Research and Consulting (CR&C) 2006); (Nay et al. 2004; Courtney et al. 2007)) and the remaining three indicator sets developed for residential aged care contained PQIs (Centres for Medicare and Medicaid Services & USA: www.cms.gov Accessed [February 2014; The American Geriatrics Society 2012; Rancourt et al. 2004).

Three indicator sets are currently systematically externally reported (National Committee for Quality Assurance & USA: www.ncqa.org Accessed [September 2013; Centres for Medicare and Medicaid Services & USA: www.cms.gov Accessed [February 2014; Primary Care & Social Care Information Centre 2013). Sixteen of the indicator sets have or are undergoing further validation studies and/or subsets of their dataset have been reported elsewhere (Nay et al. 2004; Courtney et al. 2007; Wegner et al. 2007; Basger et al. 2008; Hilmer et al. 2007; Holt et al. 2010; McLeod et al. 1997; The American Geriatrics Society 2012; Hanlon et al. 1992; Rognstad et al. 2009; Huisman-Baron et al. 2011; O'Mahony et al. 2010; Laroche et al. 2007; Rancourt et al. 2004; Onder et al. 2013; Robertson & MacKinnon 2002). No evidence of further validation, use or reporting was found for six indicator sets (Australian Institute of Health and Welfare 2009; Department of Health 2012; Campbell Research and Consulting (CR&C) 2006; NSW Therapeutic Assessment Group 2007; National Prescribing Service 2006; Winit-Watjana et al. 2008).

### Content analysis

The psychometric properties of the identified indicators sets are listed in Table 2. Nineteen (76%) of the indicator sets contained only process indicators (Campbell Research and Consulting (CR&C) 2006; Nay et al. 2004; Courtney et al. 2007; Centres for Medicare and Medicaid Services & USA: www.cms.gov Accessed [February 2014; Wegner et al. 2007; NSW Therapeutic Assessment Group 2007; Basger et al. 2008; Hilmer et al. 2007; Holt et al. 2010; The American Geriatrics Society 2012; Hanlon et al. 1992; Rognstad et al. 2009; Huisman-Baron et al. 2011; O'Mahony et al. 2010; Winit-Watjana et al. 2008; Laroche et al. 2007; Rancourt et al. 2004; Onder et al. 2013; Naugler et al. 2000) and the majority of these (n = 13) were PQIs. Only one indicator set included medication-related outcome indicators, the preventable drug-related morbidity (PDRM) indicator set, which had over fifty medication-related outcome indicators (Mackinnon & Hepler 2002). Five sets contained a combination of structural, process and outcome indicators (National Committee for Quality Assurance & USA: www.ncqa.org Accessed [September 2013; Australian Institute of Health and Welfare 2009; Department of Health 2012; Primary Care & Social Care Information Centre 2013; National Prescribing Service 2006). The majority (80%) included only explicit indicators (National Committee for Quality Assurance & USA: www.ncqa.org Accessed [September 2013; Australian Institute of Health and Welfare 2009; Campbell Research and Consulting (CR&C) 2006; Nay et al. 2004; Courtney et al. 2007; Centres for Medicare and Medicaid Services & USA: www.cms.gov Accessed [February 2014; Wegner et al. 2007; Primary Care & Social Care Information Centre 2013; NSW Therapeutic Assessment Group 2007; National Prescribing Service 2006; Mackinnon & Hepler 2002; Hilmer et al. 2007; Holt et al. 2010; The American Geriatrics Society 2012; Rognstad et al. 2009; O'Mahony et al. 2010; Winit-Watjana et al. 2008; Laroche et al. 2007; Rancourt et al. 2004; Naugler et al. 2000) and 92% were developed through extensive literature reviews and expert consensus methods, which implies both face and content validity. Common methodologies used to achieve consensus were the Delphi method, which uses consecutive rounds of anonymous consultation to achieve group consensus, and expert panels. Reliability was tested in seven of the indicator sets (National Committee for Quality Assurance & USA: www.ncqa.org Accessed [September 2013; Centres for Medicare and Medicaid Services & USA: www.cms.gov Accessed [February 2014; NSW Therapeutic Assessment Group 2007; National Prescribing Service 2006; The American Geriatrics Society 2012; Hanlon et al. 1992; Naugler et al. 2000). Feasibility, usually in the form of piloting was undertaken for eight of the indicator sets (National Committee for Quality Assurance & USA: www.ncqa.org Accessed [September 2013; Courtney et al. 2007; NSW Therapeutic Assessment Group 2007; National Prescribing Service 2006; The American Geriatrics Society 2012; Hanlon et al. 1992; O'Mahony et al. 2010; Naugler et al. 2000). Consumers were involved in the development process for seven of the indicator sets (Australian Institute of Health and Welfare 2009; Department of Health 2012; Campbell Research and Consulting (CR&C) 2006; Nay et al. 2004; Centres for Medicare and Medicaid Services & USA: www.cms.gov Accessed [February 2014; Primary Care & Social Care Information Centre 2013; National Prescribing Service 2006) five of which were Australian.

**Table 2:** **Content analysis results for identified indicator sets**

**Initial validation** (Please note references may refer to original article or article(s) discussing subsequent validation)	
**Key characteristic**	**Number (%)**
Health care aspect (structural, process and outcome)*	19 (76%) process (Campbell Research and Consulting (CR&C) (Campbell Research and Consulting (CR&C) 2006; Nay et al. 2004; Courtney et al. 2007; Wegner et al. 2007; NSW Therapeutic Assessment Group 2007; Basger et al. 2008; Hilmer et al. 2007; Holt et al. 2010; Hanlon et al. 1992; Rognstad et al. 2009; Huisman-Baron et al. 2011; O'Mahony et al. 2010; Winit-Watjana et al. 2008; Laroche et al. 2007; Rancourt et al. 2004; Onder et al. 2013; Naugler et al. 2000))
	1 (4%) outcome (Mackinnon & Hepler 2002)
	5 (20%) combination (National Committee for Quality Assurance & USA: www.ncqa.org Accessed [September 2013; Australian Institute of Health and Welfare 2009; Department of Health 2012; Primary Care & Social Care Information Centre 2013; National Prescribing Service 2006)
Explicit or implicit*	20(80%) explicit ((National Committee for Quality Assurance & USA: www.ncqa.org Accessed [September 2013; Australian Institute of Health and Welfare 2009); Campbell Research and Consulting (CR&C) (Campbell Research and Consulting (CR&C) 2006; Nay et al. 2004; Courtney et al. 2007; Centres for Medicare and Medicaid Services & USA: www.cms.gov Accessed [February 2014; Wegner et al. 2007; Primary Care & Social Care Information Centre 2013; NSW Therapeutic Assessment Group 2007; National Prescribing Service 2006; Mackinnon & Hepler 2002; Hilmer et al. 2007; Holt et al. 2010; The American Geriatrics Society 2012; Rognstad et al. 2009; O'Mahony et al. 2010; Winit-Watjana et al. 2008; Laroche et al. 2007; Rancourt et al. 2004; Naugler et al. 2000))
	3 (12%) implicit (Department of Health 2012; Hanlon et al. 1992; Huisman-Baron et al. 2011)
	2 (8%) combination(Basger et al. 2008; Onder et al. 2013)
Validity and reliability	23 (92%) developed via literature review and consensus methods except DBI (Hilmer et al. 2007) and the Australian Prescribing Indicators Tool (Basger et al. 2008)
	7 (28%) tested for reliability (National Committee for Quality Assurance & USA: www.ncqa.org Accessed [September 2013; Centres for Medicare and Medicaid Services & USA: www.cms.gov Accessed [February 2014; NSW Therapeutic Assessment Group 2007; National Prescribing Service 2006; The American Geriatrics Society 2012; Hanlon et al. 1992; Naugler et al. 2000)
Real life application (transferability and feasibility)	8 (32%) tested for feasibility (National Committee for Quality Assurance & USA: www.ncqa.org Accessed [September 2013; Courtney et al. 2007; NSW Therapeutic Assessment Group 2007; National Prescribing Service 2006; The American Geriatrics Society 2012; Hanlon et al. 1992; O'Mahony et al. 2010; Naugler et al. 2000)
Consumer involvement	7 (28%)((Australian Institute of Health and Welfare 2009; Department of Health 2012); Campbell Research and Consulting (CR&C) (Campbell Research and Consulting (CR&C) 2006; Nay et al. 2004; Centres for Medicare and Medicaid Services & USA: www.cms.gov Accessed [February 2014; Primary Care & Social Care Information Centre 2013; National Prescribing Service 2006))
**Subsequent validation** (post development)	
Validity	5 (20%) tested for predictive validity (Spinewine et al. 2007; Hamilton et al. 2011; Wilson et al. 2012; Frazier 2005)
	5 (20%) tested for concurrent validity (Hamilton et al. 2011; Luo et al. 2012)
Real life application (external validity and feasibility)	6 (24%) used in different countries or setting (Gallagher et al. 2011; Askari et al. 2012; Hanlon & Schmader 2013; Fiona 2011)
	3 (12%) routinely externally reported (National Committee for Quality Assurance & USA: www.ncqa.org Accessed [September 2013; Centres for Medicare and Medicaid Services & USA: www.cms.gov Accessed [February 2014; Primary Care & Social Care Information Centre 2013)

(*refers to the medication-related quality of care indicators only).

Many of the indicator sets have been further validated through application in research and reporting. Five indicators sets have been specifically assessed for their association (predictive validity) with health outcomes. These are the Beer’s criteria, Inappropriate Prescribing in the Elderly Tool (IPET), Medication Appropriateness Index (MAI), Drug Burden Index (DBI) and the Screening Tool of Older Person’s Prescriptions (STOPP) indicators. A review of Beer’s criteria, IPET and MAI found results ranging from a positive association with a higher risk of death, adverse drug reactions and health service utilisation to no association with mortality and hospital admissions (Spinewine et al. 2007). STOPP indicators have recently been significantly associated with preventable medication-related hospitalizations (Hamilton et al. 2011). No association between the DBI and mortality in older residential aged care residents has been reported (Wilson et al. 2012). Polypharmacy is used as an indicator of medication-related quality of care in several of the identified indicator sets and this has been associated with an increased risk of adverse events such as hospitalisation and death (Frazier 2005).

Concurrent validity has been examined for five indicator sets. A study compared the sensitivity and feasibility of the Beer’s criteria, IPET, Healthcare Effectiveness Data and Information Set (HEDIS) and the MAI and demonstrated an inverse relationship between ease of application (in terms of time and resources) and comprehensiveness (Luo et al. 2012). The MAI was found to be the most comprehensive and time consuming. HEDIS was reported as the simplest approach but less sensitive to detecting inappropriate prescribing (Luo et al. 2012). A study comparing the STOPP and Beer’s criteria found that the STOPP criteria were more sensitive to detecting potentially inappropriate medications and associated hospital admissions than Beer’s criteria (Hamilton et al. 2011).

In terms of external validity, the Beer’s criteria, STOPP/Screening Tool to Alert to Right Treatment (START) criteria, Assessing Care of Vulnerable Elders (ACOVE) indicators and MAI have been applied to many health care settings (often with minor adaptations) and in different countries with varying results (Gallagher et al. 2011; Askari et al. 2012; Hanlon & Schmader 2013). The Quality and Outcomes Framework indicators were applied to aged care residents (Fiona 2011) and found poor quality of care.

The three indicator sets which are routinely externally reported (Minimum Dataset (MDS), HEDIS and Quality and Outcomes Framework) are reviewed regularly for clinical validity and reliability (National Committee for Quality Assurance & USA: www.ncqa.org Accessed [September 2013; Centres for Medicare and Medicaid Services & USA: www.cms.gov Accessed [February 2014; Primary Care & Social Care Information Centre 2013) and demonstrate feasibility on a large scale.

### Relevance to medication-related quality of care needs for Australian residential aged care (minimum indicator set)

Presence of indicators which address one or more of the pre-determined six medication-related attributes is shown in Table 3, for each of the 25 indicator sets. Approximately half of the sets (n = 12) addressed medication use in the most prevalent chronic diseases (National Committee for Quality Assurance & USA: www.ncqa.org Accessed [September 2013; Australian Institute of Health and Welfare 2009; Wegner et al. 2007; Primary Care & Social Care Information Centre 2013; NSW Therapeutic Assessment Group 2007; National Prescribing Service 2006; Mackinnon & Hepler 2002; Basger et al. 2008; The American Geriatrics Society 2012; O'Mahony et al. 2010; Onder et al. 2013; Naugler et al. 2000). Twenty four indicator sets addressed general medication appropriateness and eight addressed (directly or indirectly) detection of medication-related adverse events, namely falls (National Committee for Quality Assurance & USA: www.ncqa.org Accessed [September 2013; Australian Institute of Health and Welfare 2009; Campbell Research and Consulting (CR&C) 2006; Courtney et al. 2007; Centres for Medicare and Medicaid Services & USA: www.cms.gov Accessed [February 2014; Wegner et al. 2007; NSW Therapeutic Assessment Group 2007; Mackinnon & Hepler 2002). Seven assessed medication-related services (National Committee for Quality Assurance & USA: www.ncqa.org Accessed [September 2013; Australian Institute of Health and Welfare 2009; Department of Health 2012; Courtney et al. 2007; Wegner et al. 2007; Primary Care & Social Care Information Centre 2013; National Prescribing Service 2006) and four addressed medication policy (Department of Health 2012; Wegner et al. 2007; Primary Care & Social Care Information Centre 2013; National Prescribing Service 2006). Two indicator sets (Assessing Care of Vulnerable Elders (ACOVE) and CRIteria to assess appropriate Medication use among Elderly complex patients (CRIME)) addressed review of medication with respect to limited life expectancy (O'Mahony et al. 2010; Onder et al. 2013).

**Table 3:** **Mapping of identified indicator sets to core medication-related criteria**

**Name of indicator/indicator set**	**Medication-related quality of care attributes relevant to residential aged care in Australia (+ = present)**					
	**Medication appropriateness for the most prevalent chronic diseases**	**Medication appropriateness in limited life expectancy**	**General medication appropriateness**	**Detection & monitoring of adverse drug events**	**Access to services (e.g. medication review and care plans)**	**Medication-related policy and procedures**
Resident-centred quality indicators in residential aged care or The Campbell Report (Campbell Research and Consulting (CR&C) (Campbell Research and Consulting (CR&C) 2006))	**-**	**-**	**+**	**+**	**-**	**-**
Public Sector Residential Aged Care Quality of Care Performance Indicators (Nay et al. 2004)	**-**	**-**	**+**	**+**	**-**	**-**
Clinical Care Indicators Tool or CCI or Uniting Care Clinical Care Indicators (Courtney et al. 2007; Courtney et al. 2010)	**-**	**-**	**+**	**+**	**+**	**-**
National indicators of safety and quality in health care (Australian Institute of Health and Welfare 2009)	**+**	**-**	**+**	**+**	**+**	**-**
Minimum Data Set (version 3.0) Nursing Home Quality measures (Centres for Medicare and Medicaid Services & USA: www.cms.gov Accessed [February 2014; Zimmerman et al. 1995; Hawes et al. 1997)	**-**	**-**	**+**	**+**	**-**	**-**
Assessing Care of Vulnerable Elders (version 3) or ACOVE–3 (Wegner et al. 2007) (USA)	**+**	**-**	**+**	**+**	**+**	**+**
Healthcare effectiveness data and information set or HEDIS (National Committee for Quality Assurance & USA: www.ncqa.org Accessed [September 2013)	**+**	**-**	**+**	**+**	**+**	**-**
Quality and Outcomes Framework or QOF (Primary Care & Social Care Information Centre 2013)	**+**	**-**	**+**	**-**	**+**	**+**
Guiding principles for medication management in residential aged care facilities (Department of Health 2012)	**-**	**-**	**-**	**-**	**+**	**+**
Indicators for Quality Use of Medicines in Australian Hospitals (NSW Therapeutic Assessment Group 2007)	**+**	**-**	**+**	**-**	**-**	**-**
Indicators for Quality Prescribing in Australian General Practice (National Prescribing Service 2006)	**+**	**-**	**+**	**-**	**+**	**+**
Preventable Drug Related Morbidity (PDRM) (Mackinnon & Hepler 2002; Robertson & MacKinnon 2002; Morris et al. 2002)	**+**	**-**	**+**	**+**	**-**	**-**
Australian Prescribing Indicators Tool (Basger et al. 2008)	**+**	**-**	**+**	**-**	**-**	**-**
Drug Burden Index or DBI (Hilmer et al. 2007)	**-**	**-**	**+**	**-**	**-**	**-**
The PRISCUS List (Holt et al. 2010)	**-**	**-**	**+**	**-**	**-**	**-**
Inappropriate Prescribing in the Elderly Tool or IPET (McLeod et al. 1997; Naugler et al. 2000)	**+**	**-**	**+**	**-**	**-**	**-**
Beer’s criteria (The American Geriatrics Society 2012)	**+**	**-**	**+**	**-**	**-**	**-**
Medication Appropriateness Index or MAI (Hanlon et al. 1992)	**-**	**-**	**+**	**-**	**-**	**-**
NORGEP criteria for assessing inappropriate prescriptions to elderly patients (Rognstad et al. 2009)	**-**	**-**	**+**	**-**	**-**	**-**
Criteria for drug selection in frail elderly patients (Huisman-Baron et al. 2011)	**-**	**-**	**+**	**-**	**-**	**-**
Screening Tool of Older Person’s Prescriptions (STOPP) and Screening Tool to Alert to Right Treatment (START) (O'Mahony et al. 2010; Barry et al. 2007; Gallagher & O'Mahony 2008)	**+**	**+**	**+**	**-**	**-**	**-**
Criteria for high-risk medication use (Winit-Watjana et al. 2008)	**-**	**-**	**+**	**-**	**-**	**-**
Potentially inappropriate medications in the elderly: a French consensus panel (Laroche et al. 2007)	**-**	**-**	**+**	**-**	**-**	**-**
Potentially Inappropriate Prescriptions for older patients in long-term care or PIP (Rancourt et al. 2004)	**-**	**-**	**+**	**-**	**-**	**-**
CRIteria to assess appropriate Medication use among Elderly complex patients or CRIME criteria (Onder et al. 2013)	**+**	**+**	**+**	**-**	**-**	**-**

Table 4 shows the selected indicators, derived from the mapping results in Table 3, to define a minimum indicator set for evaluating medication-related care in Australian residential aged care. In total 28 individual indicators were identified which address five of the six attributes. Twenty two of the 25 indicator sets are represented in the minimum dataset.

**Table 4:** **Minimum indicator set for evaluating medication-related quality of care in Australian residential aged care**

**Core criteria addressed**	**Indicator**
1. Medication appropriateness in the most prevalent diseases	1.1 Use of beta blocker post myocardial infarction/ischaemic heart disease (National Committee for Quality Assurance & USA: www.ncqa.org Accessed [September 2013; Australian Institute of Health and Welfare 2009; Wegner et al. 2007; Primary Care & Social Care Information Centre 2013; NSW Therapeutic Assessment Group 2007; National Prescribing Service 2006; Basger et al. 2008)
	1.2 Use of statin post myocardial infarction/ischaemic heart disease (Australian Institute of Health and Welfare 2009; Wegner et al. 2007; Primary Care & Social Care Information Centre 2013; NSW Therapeutic Assessment Group 2007; National Prescribing Service 2006; O'Mahony et al. 2010)
	1.3 Angiotensin converting enzyme inhibitor/angiotensin receptor blocker use in hypertension/congestive heart failure (Wegner et al. 2007; Primary Care & Social Care Information Centre 2013; NSW Therapeutic Assessment Group 2007; National Prescribing Service 2006; Mackinnon & Hepler 2002; Basger et al. 2008; O'Mahony et al. 2010)
	1.4 Antiplatelet therapy post myocardial infarction/ischaemic heart disease (e.g. aspirin/clopidogrel not ticlopidine) (National Committee for Quality Assurance & USA: www.ncqa.org Accessed [September 2013; Australian Institute of Health and Welfare 2009; Wegner et al. 2007; Primary Care & Social Care Information Centre 2013; NSW Therapeutic Assessment Group 2007; National Prescribing Service 2006; Basger et al. 2008)
	1.5 Antiplatelet therapy post stroke/transient ischaemic attacks (e.g. aspirin/clopidogrel not ticlopidine) (Wegner et al. 2007; Primary Care & Social Care Information Centre 2013; NSW Therapeutic Assessment Group 2007; Basger et al. 2008; O'Mahony et al. 2010)
	1.6 Medicines to avoid in patients with cardiovascular disease (e.g. NSAIDs/COX 2 inhibitors, calcium channel blockers and select anti-arrhythmics) (Wegner et al. 2007; Mackinnon & Hepler 2002; Basger et al. 2008; The American Geriatrics Society 2012; O'Mahony et al. 2010; Winit-Watjana et al. 2008; Naugler et al. 2000)
	1.7 Medicines to avoid in patients with dementia (e.g. medications with clinically significant anticholinergic properties) (Wegner et al. 2007; Basger et al. 2008; The American Geriatrics Society 2012; O'Mahony et al. 2010; Laroche et al. 2007; Onder et al. 2013)
2. Medication appropriateness in limited life expectancy	Did not meet inclusion criteria
3. General medication appropriateness	3.1 Medicines to avoid in patients at risk of falling or with a history of falls ( e.g. medications with clinically significant anticholinergic properties, sedating antihistamines, tricyclic antidepressants, monoamine oxidase inhibitors, selective serotonin reuptake inhibitors, benzodiazepines and antipsychotics) (Wegner et al. 2007; Mackinnon & Hepler 2002; Basger et al. 2008; The American Geriatrics Society 2012; Rognstad et al. 2009; O'Mahony et al. 2010)
	3.2 Avoid use of benzodiazepines (short and long acting) (Wegner et al. 2007; National Prescribing Service 2006; Basger et al. 2008; Holt et al. 2010; The American Geriatrics Society 2012; Rognstad et al. 2009; O'Mahony et al. 2010; Winit-Watjana et al. 2008; Laroche et al. 2007; Rancourt et al. 2004; Naugler et al. 2000)
	3.3 Avoid use of medicines with clinically significant anticholinergic properties (Wegner et al. 2007; Basger et al. 2008; Holt et al. 2010; The American Geriatrics Society 2012; Rognstad et al. 2009; O'Mahony et al. 2010; Winit-Watjana et al. 2008; Laroche et al. 2007; Naugler et al. 2000)
	3.4 Anti-arrhythmic medicines to avoid (e.g. disopyramide and see 1.6) (The American Geriatrics Society 2012; Rognstad et al. 2009; Laroche et al. 2007; Naugler et al. 2000)
	3.5 Digoxin > 0.125mcg/day (The American Geriatrics Society 2012; O'Mahony et al. 2010; Winit-Watjana et al. 2008; Laroche et al. 2007; Rancourt et al. 2004; Naugler et al. 2000)
	3.6 Antidepressants to avoid (e.g. tricyclic antidepressants (see 3.1 and 3.3) and monoamine oxidase inhibitors (see 3.1) (Wegner et al. 2007; Holt et al. 2010; The American Geriatrics Society 2012; Rognstad et al. 2009; O'Mahony et al. 2010; Winit-Watjana et al. 2008; Rancourt et al. 2004; Naugler et al. 2000)
	3.7 Avoid typical (see 3.3) and atypical antipsychotics (e.g. olanzapine and clozapine) (Holt et al. 2010; The American Geriatrics Society 2012; Rognstad et al. 2009; O'Mahony et al. 2010; Winit-Watjana et al. 2008; Rancourt et al. 2004)
	3.8 Antispasmodics and muscle relaxants to avoid ( smooth muscle relaxants alverine and mebeverine and see 3.3) (McLeod et al. 1997; Moher et al. 2009; National Aged Care Alliance 2014; National Committe for Quality Assurance. (Desirable attributes of HEDIS). USA: www.ncqa.org/tabid/415/Default.aspx Accessed [March 2012; National Committee for Quality Assurance & USA: www.ncqa.org Accessed [September 2013; National Institute for Health and Care Excellence 2014a; Onder et al. 2013)
	3.9 Avoid duplication of drug class (e.g. >2 NSAIDs) (Mackinnon & Hepler 2002; O'Mahony et al. 2010; Winit-Watjana et al. 2008; Laroche et al. 2007; Rancourt et al. 2004)
	3.10 Avoid alpha blockers (e.g. prazosin and doxazosin) (Holt et al. 2010; The American Geriatrics Society 2012; O'Mahony et al. 2010; Winit-Watjana et al. 2008)
	3.11 Avoid centrally acting alpha agonists (e.g. clonidine and methyldopa) (Holt et al. 2010; The American Geriatrics Society 2012; Laroche et al. 2007; Rancourt et al. 2004; Naugler et al. 2000)
	3.12 Calcium channel blockers to avoid (e.g. short acting nifedipine) (Holt et al. 2010; The American Geriatrics Society 2012; Winit-Watjana et al. 2008; Laroche et al. 2007; Naugler et al. 2000)
	3.13 Avoid combination of warfarin and aspirin (+/− gastric protection) (Rognstad et al. 2009; O'Mahony et al. 2010; Winit-Watjana et al. 2008; Rancourt et al. 2004)
	3.14 Avoid ‘Triple Whammy’ combination of angiotensin converting enzyme inhibitor/angiotensin two receptor antagonist plus diuretic plus non-steroidal anti-inflammatory (excluding low dose aspirin) (National Prescribing Service 2006; Basger et al. 2008; Rognstad et al. 2009)
	3.15 Influenza vaccination rates (National Committee for Quality Assurance & USA: www.ncqa.org Accessed [September 2013; Australian Institute of Health and Welfare 2009; Centres for Medicare and Medicaid Services & USA: www.cms.gov Accessed [February 2014; Wegner et al. 2007; Primary Care & Social Care Information Centre 2013; Basger et al. 2008)
	3.16 Pneumococcal vaccinations rates (National Committee for Quality Assurance & USA: www.ncqa.org Accessed [September 2013; Australian Institute of Health and Welfare 2009; Centres for Medicare and Medicaid Services & USA: www.cms.gov Accessed [February 2014; Wegner et al. 2007; Basger et al. 2008)
4. Detection and monitoring of adverse events	4.1 Fall rates (+/− associated with medication use) (Australian Institute of Health and Welfare 2009; Campbell Research and Consulting (CR&C) 2006; Nay et al. 2004; Courtney et al. 2007; Centres for Medicare and Medicaid Services & USA: www.cms.gov Accessed [February 2014; Wegner et al. 2007; Mackinnon & Hepler 2002; Onder et al. 2013)
5. Access to services	5.1 Annual cycle of care for people with chronic disease (e.g. diabetes) (National Committee for Quality Assurance & USA: www.ncqa.org Accessed [September 2013; Australian Institute of Health and Welfare 2009; Wegner et al. 2007; Primary Care & Social Care Information Centre 2013)
	5.2 Medication review (National Committee for Quality Assurance & USA: www.ncqa.org Accessed [September 2013; Australian Institute of Health and Welfare 2009; Department of Health 2012; Courtney et al. 2007; Wegner et al. 2007; Primary Care & Social Care Information Centre 2013; National Prescribing Service 2006)
6. Policy/Procedure	6.1 Access to up to date medicines information for providers, carers and residents (Department of Health 2012; Wegner et al. 2007; National Prescribing Service 2006)
	6.2 Policy of regular medication review including over the counter and complementary medicines (Department of Health 2012; Wegner et al. 2007; Primary Care & Social Care Information Centre 2013; National Prescribing Service 2006)

## Discussion

In this study we identified 25 sets of medication-related quality of care indicators relevant to residential aged care. From these, 28 indicators that address key medication-related quality of care issues specific for residential aged care were selected to form the minimum indicator set. All 28 indictors have been previously validated for face and content validity through either extensive literature reviews and or expert consensus. Furthermore, external validity was demonstrated across countries for some of these indicators. Indicators for assessment of medication appropriateness, PQIs, were common among the identified indicators sets which demonstrated a strong international consensus on the appropriateness of these medications use in the elderly. Indicators for addressing the medication appropriateness for the most prevalent diseases, detecting and monitoring adverse drug events, access to relevant services and medication-related policy were identified in at least three of the included indicators sets. Indicators to address medication use in limited life expectancy in aged care residents are lacking.

Two of the identified indicator sets developed specifically for aged care take quite an extensive approach to medication management. ACOVE-3 contains nearly 400 indicators with one quarter of the indicators related to medication use (Wegner et al. 2007). This indicator set addresses prescribing, monitoring of medicines and consumer involvement in medication management. It has been extended to several subsets of indicators such as quality of care in dementia and poor prognosis. The Australian Guiding Principles for Medication Management in Residential Aged Care has a broad approach to evaluating medication use ranging from reviewing medicines policies and procedures to annual medication review but does not target specific medications (Department of Health 2012). Twelve of the indicators in our minimum indicator set are from ACOVE-3 and three are from the Australian Guiding Principles for Medication Management in Residential Aged Care. This demonstrates the relevance of these indicators for evaluating medication-related quality of care in Australian residential aged care. Whilst ACOVE-3 is a very comprehensive indicator set, it is burdensome for implementing in a clinical setting with 98 indicators relating to medication-related quality of care, many of which address clinical conditions not prevalent in the residential Australian aged care population. Currently quality of care indicator sets developed specifically for Australian residential aged care include simplistic medication-related quality of care indicators such as polypharmacy and medication review (Table 1). These two indicators are useful tools for identifying residents at risk of inappropriate medication-related quality of care and predicting adverse health outcomes however they do not reflect the complexity of medication-related care for the residential aged care population (Duerden et al. 2013). The minimum indicator set developed in this study provides a platform for a quality of care indicator set specific for Australian residential aged care that focuses on the key requirements to examine medication-related quality of care in this setting.

The overwhelming majority of medication-related quality of care indicator sets identified included exclusively PQIs. PQIs play an important role in evaluating medication-related quality of care. They are evidence based and specifically target medications known to cause adverse effects. Furthermore, the PQI sets developed for the older age groups address medicines most problematic in this population. PQI sets are often criticised due to their lack of sensitivity as they are designed to be generically applied across a population and do not consider variability in individual patient medical needs and preferences (Spinewine et al. 2007; Steinman et al. 2007). This however enhances the feasibility in reporting of these indicators. These qualities of PQIs are reflected by their prevalence in the minimum indicator set. Some PQI sets target medications to be avoided in certain comorbid states (e.g. Beer's criteria) and others (e.g. CRIME) addressed medication use in clinically complex frail elderly patients (Onder et al. 2013).

This review identified indicator sets which involved consumers in the development, however an indicator set with a more holistic approach to medication-management in the frail elderly with respect to prioritizing patient preferences and quality of life (patient-centred care) was not identified. The patient-centred care approach aligns with the current approach to delivering quality health care services in many countries, including Australia (Institute of Medicine. Committee on Quality of Health Care in America 2001; Australian Commission on Safety and Quality in Health Care 2010). Development of medication-related quality of care indicators with respect to patient-centred care warrants further consideration.

Collection and analysis of data for reporting quality of care indicators can be resource intensive. Experts in performance measurement in health care rate feasibility, scientific soundness and relevance as the three most desirable attributes for indicators (National Committe for Quality Assurance. (Desirable attributes of HEDIS). USA: www.ncqa.org/tabid/415/Default.aspx Accessed [March 2012). All of the indictors in the minimum indicator set are operational in that they are currently used in research and or quality of care reporting systems

### Study limitations

The potential for bias in identification and selection of articles was limited by strictly adhering to predefined inclusion and exclusion criteria and group consensus on differences. The PRISMA guidelines were used in both the study methodology and reporting of the results however, we cannot rule out that relevant studies or reports have been missed. We also used the CASP criteria to ensure that all included articles were of a minimum quality. Two authors (AV and GC) were deemed suitable to oversee and contribute to all steps of this study as they are experienced in undertaking and publishing systematic reviews (Spurling et al. 2010; Caughey et al. 2008).

We also chose a systematic approach to the data extraction using predefined criteria to minimise bias in reporting of the qualities of each identified indicator set. Content analysis methodology has been criticised for being too reductive and consequently we may have missed identifying important indicator qualities (Dixon-Woods et al. 2005).

Every effort was made to correctly designate operational status to each indicator set. However, many of the indicator sets are intended for internal quality monitoring and reporting and it is possible that some of the indicators are reported in house or are operational through incorporation into other datasets.

Only five of the indicators sets identified have been formally assessed for predictive validity (with varying results). Further research should focus on investigating the relationships between our developed minimum indicator set and health outcomes.

Whilst the identified indicators in our minimum indicator set have content and face validity for medication-related quality of care in the elderly, many are yet to be validated for the Australian aged care setting. Indicators for assessing access to medication-related health services were not common and may reflect the differences in health systems between countries. Indicators addressing medication-related health services specific to residential aged care residents in Australia, would need to be further investigated for inclusion in the minimum indicator set. Subsequent development and validation of the minimum indicator set should be the focus of future research.

## Conclusion

In this study we have derived a minimum indicator set for evaluating medication-related quality of care in residential aged care using systematically identified and previously validated indicators based on the key medication-related needs of the aged care population. The developed minimum indicator set is intended as a starting point for comprehensively evaluating medication-related quality of care in Australian aged care. This study identified that indicators focusing on pertinent medication-related issues for older aged care residents, namely patient-centred care and limited life expectancy, were lacking and warrant further attention. Also, inclusion of additional indicators which address detection and monitoring of adverse events, not just falls, as these were not overly prevalent in the indicator sets. The focus on the core medication-related attributes in the minimum indicator set improves the feasibility of implementation in the aged care setting by minimising data collection and management. Validation of the minimum indicator set in the Australian aged care setting will provide a means to assess and monitor quality of care in this growing health sector.
